# Supplementary material for: Understanding motivations of older women to continue or discontinue breast cancer screening
Source: PLoS One. 2025 Jun 5;20(6):e0319141. doi: 10.1371/journal.pone.0319141 (PMC12140224; doi:10.1371/journal.pone.0319141)
Supplement: S1 Table — (DOCX) [file pone.0319141.s001.docx]

**SUPPORTING INFORMATION**

**TABLE: PROTOCOL: OPEN-ENDED QUESTIONS ABOUT SCREENING**

| **Type** | **Question** |
| --- | --- |
| General information about mammograms | What is a mammogram? |
|  | What are they used for? |
|  | What are some of the benefits? |
|  | What are some of the risks? |
| The decision process | Can you tell me about the most recent time you thought about having a mammogram? |
|  | Who initiated the decision? |
|  | How much involvement do you want your doctor to have in your decision? |
|  | Do you intend to continue getting mammograms? |
|  | When was your most recent mammogram? |
