# Supplementary material for: Understanding motivations of older women to continue or discontinue breast cancer screening
Source: PLoS One. 2025 Jun 5;20(6):e0319141. doi: 10.1371/journal.pone.0319141 (PMC12140224; doi:10.1371/journal.pone.0319141)
Supplement: S2 Table — (DOCX) [file pone.0319141.s002.docx]

**VARIABLE LIST (59 RESPONDENTS X 29 VARIABLES)**

1.ID2 = ID number renumbered in order in file

2.ID1 = Original study ID numbers

3.Age = Age in Years

4.AgeD = Age (0) 70-74 years of age, (1) 75+ years of age

5.Race = Race (1) White, (2) African-American, (3) Hispanic

6.EducD = Education (0) high school or less, (1) more than high school

7.HealthR = Self-rated health (1) poor

Description (0=no, not mentioned; 1=yes, mentioned; 99 missing)

8.Xray = Breast X-Ray

9.Image = Breast Image

10.Exam = Breast Exam

Purpose (0=no, not mentioned; 1=yes, mentioned; 99 missing)

11.DetCA = Detect cancer

12.DetLump = Detect Lump

13.DetAbn = Detect Abnormality

Benefits (0=no, not mentioned; 1=yes, mentioned; 99 missing)

14.BenCA = Detect cancer

15.BenLump = Detect lump/abnormality

16.EarlyTX = Timely treatment

17.Live = Live longer

18.Health = Health reassurance

Risks (0=no, not mentioned; 1=yes, mentioned; 99 missing)

19.Risk0 = No risk

20.RiskRad = Radiation/cancerous

21.RiskPain = Pain or discomfort

22.RiskInacc = Inaccurate reading

Decision Process (0=no, not mentioned; 1=yes, mentioned; 99 missing)

23.InitSelf = Self-Initiated screening

24.InitDr = Doctor initiated screening/scheduling

25.Remin = Reminder

26.FamCA = Personal cancer story

27.DrYes = Doctor recommended screening (verbally)

28.RecentM = Recent screening, screening within past three years

29.Continue = Intent to (0) Discontinue or (1) Continue

| 1  ID2 | 2  ID1 | 3  Age | 4AgeD | 5Race | 6  EducD | 7  HealthR | 8  Xray | 9  Image | 10  Exam | 11  DetCa | 12  Detlump | 13  DetAbn | 14  BenCA | 15  BenLump | 16  EarlyTx | 17  Live | 18  Health | 19  Risk0 | 20  RiskRad | 21  RiskPain | 22  RiskInacc | 23  InitSelf | 24  InitDr | 25  Remin | 26  FamCA | 27  DrYes | 28  RecentM | 29  CONTINUE |
| --- | --- | --- | --- | --- | --- | --- | --- | --- | --- | --- | --- | --- | --- | --- | --- | --- | --- | --- | --- | --- | --- | --- | --- | --- | --- | --- | --- | --- |
| 1 | 4 | 78 | 1 | 1 | 0 | 3 | 99 | 99 | 99 | 0 | 0 | 1 | 0 | 0 | 1 | 0 | 0 | 0 | 0 | 1 | 0 | 0 | 1 | 0 | 1 | 1 | 1 | 1 |
| 2 | 5 | 73 | 0 | 2 | 1 | 3 | 0 | 1 | 0 | 1 | 0 | 0 | 0 | 0 | 1 | 1 | 0 | 0 | 0 | 0 | 1 | 0 | 1 | 0 | 1 | 1 | 1 | 1 |
| 3 | 6 | 83 | 1 | 1 | 0 | 3 | 0 | 0 | 1 | 1 | 0 | 0 | 1 | 0 | 0 | 0 | 0 | 0 | 1 | 1 | 0 | 0 | 0 | 0 | 1 | 0 | 0 | 0 |
| 4 | 7 | 72 | 0 | 3 | 1 | 3 | 0 | 1 | 0 | 0 | 1 | 0 | 0 | 0 | 1 | 0 | 0 | 1 | 0 | 0 | 0 | 1 | 0 | 1 | 0 | 1 | 1 | 1 |
| 5 | 8 | 92 | 1 | 1 | 1 | 4 | 1 | 0 | 0 | 0 | 0 | 1 | 1 | 0 | 1 | 0 | 0 | 0 | 0 | 0 | 1 | 0 | 1 | 1 | 1 | 0 | 0 | 0 |
| 6 | 9 | 77 | 1 | 1 | 0 | 3 | 0 | 1 | 0 | 1 | 1 | 0 | 1 | 1 | 0 | 0 | 0 | 0 | 1 | 0 | 0 | 0 | 1 | 1 | 1 | 1 | 1 | 0 |
| 7 | 10 | 86 | 1 | 1 | 1 | 3 | 0 | 1 | 0 | 0 | 1 | 0 | 0 | 1 | 0 | 0 | 0 | 0 | 0 | 1 | 0 | 0 | 0 | 1 | 0 | 0 | 1 | 0 |
| 8 | 11 | 71 | 0 | 1 | 0 | 3 | 1 | 0 | 0 | 0 | 1 | 0 | 0 | 0 | 1 | 0 | 0 | 0 | 0 | 0 | 0 | 0 | 1 | 1 | 1 | 1 | 1 | 1 |
| 9 | 12 | 90 | 1 | 1 | 1 | 2 | 0 | 0 | 1 | 1 | 0 | 0 | 1 | 0 | 0 | 0 | 0 | 1 | 0 | 0 | 0 | 0 | 1 | 0 | 0 | 0 | 0 | 0 |
| 10 | 14 | 79 | 1 | 2 | 1 | 2 | 0 | 0 | 1 | 1 | 0 | 1 | 0 | 1 | 0 | 0 | 0 | 0 | 0 | 1 | 0 | 1 | 0 | 0 | 1 | 0 | 1 | 1 |
| 11 | 15 | 75 | 1 | 1 | 1 | 3 | 1 | 0 | 0 | 1 | 1 | 0 | 1 | 0 | 0 | 0 | 0 | 0 | 1 | 0 | 0 | 0 | 1 | 0 | 0 | 1 | 1 | 0 |
| 12 | 16 | 92 | 1 | 1 | 1 | 3 | 0 | 0 | 1 | 1 | 0 | 0 | 1 | 0 | 0 | 0 | 0 | 0 | 1 | 0 | 0 | 0 | 1 | 0 | 1 | 0 | 0 | 0 |
| 13 | 17 | 71 | 0 | 1 | 0 | 2 | 0 | 0 | 1 | 0 | 1 | 0 | 0 | 1 | 0 | 1 | 0 | 1 | 0 | 1 | 0 | 0 | 0 | 1 | 1 | 1 | 1 | 1 |
| 14 | 18 | 84 | 1 | 3 | 0 | 4 | 1 | 0 | 0 | 1 | 0 | 1 | 0 | 1 | 0 | 0 | 0 | 0 | 1 | 0 | 0 | 0 | 0 | 1 | 0 | 0 | 1 | 0 |
| 15 | 19 | 72 | 0 | 1 | 1 | 2 | 0 | 1 | 0 | 1 | 1 | 0 | 1 | 1 | 1 | 0 | 0 | 0 | 1 | 1 | 0 | 0 | 1 | 0 | 0 | 0 | 1 | 0 |
| 16 | 20 | 73 | 0 | 2 | 1 | 3 | 1 | 0 | 0 | 1 | 0 | 0 | 1 | 0 | 0 | 0 | 0 | 0 | 1 | 0 | 0 | 0 | 1 | 0 | 0 | 1 | 1 | 1 |
| 17 | 21 | 78 | 1 | 1 | 1 | 3 | 0 | 1 | 0 | 1 | 0 | 0 | 0 | 1 | 0 | 0 | 0 | 0 | 1 | 0 | 0 | 1 | 0 | 0 | 1 | 0 | 1 | 1 |
| 18 | 22 | 81 | 1 | 2 | 1 | 3 | 0 | 1 | 0 | 0 | 0 | 1 | 0 | 1 | 1 | 0 | 0 | 0 | 0 | 1 | 1 | 0 | 0 | 0 | 0 | 0 | 0 | 0 |
| 19 | 23 | 77 | 1 | 1 | 1 | 4 | 1 | 0 | 0 | 1 | 0 | 0 | 1 | 0 | 1 | 0 | 0 | 0 | 1 | 0 | 0 | 0 | 1 | 0 | 1 | 1 | 1 | 1 |
| 20 | 24 | 71 | 0 | 3 | 0 | 4 | 1 | 0 | 0 | 1 | 0 | 0 | 1 | 0 | 0 | 1 | 0 | 0 | 0 | 0 | 0 | 0 | 1 | 1 | 1 | 1 | 1 | 1 |
| 21 | 25 | 74 | 0 | 2 | 1 | 2 | 1 | 0 | 0 | 1 | 0 | 0 | 0 | 0 | 1 | 0 | 0 | 1 | 0 | 0 | 0 | 1 | 1 | 0 | 1 | 0 | 1 | 1 |
| 22 | 27 | 75 | 1 | 1 | 1 | 4 | 0 | 1 | 0 | 1 | 0 | 0 | 0 | 1 | 0 | 0 | 0 | 1 | 0 | 0 | 0 | 1 | 0 | 0 | 1 | 0 | 1 | 1 |
| 23 | 28 | 87 | 1 | 1 | 0 | 3 | 0 | 1 | 0 | 0 | 1 | 0 | 0 | 0 | 1 | 0 | 0 | 0 | 0 | 0 | 0 | 0 | 1 | 0 | 1 | 1 | 1 | 1 |
| 24 | 29 | 92 | 1 | 1 | 1 | 3 | 0 | 1 | 0 | 1 | 0 | 1 | 0 | 0 | 0 | 0 | 1 | 1 | 0 | 0 | 0 | 0 | 1 | 0 | 1 | 1 | 1 | 1 |
| 25 | 30 | 73 | 0 | 2 | 1 | 3 | 0 | 0 | 1 | 1 | 0 | 0 | 1 | 0 | 0 | 0 | 0 | 1 | 0 | 0 | 0 | 0 | 1 | 1 | 0 | 1 | 1 | 1 |
| 26 | 31 | 86 | 1 | 2 | 1 | 3 | 0 | 0 | 1 | 1 | 0 | 0 | 0 | 0 | 0 | 0 | 1 | 0 | 0 | 0 | 1 | 0 | 1 | 0 | 0 | 0 | 0 | 0 |
| 27 | 32 | 71 | 0 | 2 | 0 | 3 | 0 | 0 | 0 | 1 | 1 | 0 | 0 | 1 | 0 | 0 | 0 | 1 | 0 | 0 | 0 | 0 | 1 | 0 | 0 | 1 | 1 | 1 |
| 28 | 33 | 74 | 0 | 2 | 0 | 2 | 1 | 0 | 0 | 1 | 0 | 0 | 1 | 0 | 0 | 0 | 1 | 1 | 0 | 0 | 0 | 0 | 1 | 0 | 1 | 0 | 0 | 1 |
| 29 | 34 | 76 | 1 | 2 | 0 | 2 | 0 | 0 | 0 | 1 | 0 | 0 | 0 | 0 | 0 | 0 | 1 | 0 | 0 | 1 | 0 | 0 | 1 | 0 | 1 | 1 | 1 | 1 |
| 30 | 35 | 90 | 1 | 2 | 0 | 3 | 0 | 1 | 0 | 1 | 0 | 0 | 1 | 1 | 0 | 0 | 0 | 0 | 0 | 0 | 0 | 0 | 1 | 1 | 1 | 0 | 1 | 0 |
| 31 | 36 | 78 | 1 | 2 | 0 | 2 | 0 | 0 | 1 | 1 | 0 | 0 | 0 | 0 | 0 | 1 | 0 | 0 | 0 | 0 | 0 | 0 | 0 | 0 | 0 | 0 | 0 | 0 |
| 32 | 37 | 71 | 0 | 2 | 0 | 3 | 1 | 0 | 0 | 0 | 1 | 0 | 1 | 0 | 0 | 0 | 0 | 0 | 0 | 0 | 0 | 1 | 0 | 0 | 1 | 0 | 0 | 0 |
| 33 | 38 | 70 | 0 | 2 | 0 | 2 | 0 | 0 | 1 | 1 | 0 | 0 | 1 | 0 | 0 | 0 | 0 | 0 | 0 | 0 | 0 | 0 | 1 | 0 | 0 | 1 | 1 | 1 |
| 34 | 39 | 74 | 0 | 1 | 0 | 3 | 1 | 0 | 0 | 1 | 0 | 0 | 1 | 0 | 0 | 0 | 0 | 1 | 0 | 0 | 0 | 0 | 1 | 0 | 0 | 1 | 0 | 0 |
| 35 | 40 | 90 | 1 | 2 | 1 | 2 | 1 | 0 | 0 | 99 | 99 | 99 | 0 | 0 | 1 | 0 | 0 | 0 | 0 | 0 | 1 | 0 | 0 | 0 | 0 | 0 | 0 | 0 |
| 36 | 41 | 79 | 1 | 2 | 0 | 3 | 0 | 1 | 0 | 0 | 0 | 1 | 0 | 1 | 0 | 0 | 0 | 0 | 1 | 0 | 0 | 0 | 1 | 1 | 1 | 1 | 1 | 1 |
| 37 | 42 | 72 | 0 | 3 | 0 | 1 | 1 | 0 | 0 | 1 | 0 | 0 | 0 | 0 | 0 | 1 | 0 | 1 | 0 | 0 | 0 | 0 | 1 | 0 | 1 | 0 | 0 | 0 |
| 38 | 43 | 90 | 1 | 2 | 0 | 4 | 0 | 0 | 0 | 1 | 0 | 0 | 0 | 0 | 0 | 0 | 1 | 0 | 1 | 1 | 0 | 0 | 1 | 0 | 0 | 0 | 0 | 0 |
| 39 | 44 | 72 | 0 | 3 | 0 | 2 | 0 | 0 | 1 | 1 | 0 | 0 | 0 | 0 | 0 | 0 | 1 | 0 | 1 | 0 | 0 | 0 | 1 | 0 | 0 | 1 | 1 | 1 |
| 40 | 46 | 72 | 0 | 3 | 0 | 3 | 1 | 0 | 0 | 1 | 0 | 0 | 0 | 0 | 0 | 0 | 1 | 0 | 0 | 0 | 0 | 0 | 1 | 0 | 1 | 0 | 0 | 0 |
| 41 | 47 | 70 | 0 | 3 | 0 | 3 | 1 | 0 | 0 | 0 | 1 | 0 | 0 | 1 | 0 | 0 | 0 | 0 | 0 | 0 | 0 | 0 | 1 | 0 | 1 | 1 | 1 | 1 |
| 42 | 49 | 75 | 1 | 3 | 0 | 3 | 0 | 0 | 0 | 0 | 1 | 0 | 0 | 0 | 0 | 0 | 1 | 0 | 0 | 0 | 0 | 1 | 0 | 0 | 1 | 0 | 1 | 1 |
| 43 | 50 | 74 | 0 | 3 | 0 | 3 | 0 | 0 | 1 | 1 | 0 | 0 | 1 | 0 | 0 | 0 | 0 | 1 | 0 | 0 | 0 | 1 | 0 | 0 | 1 | 0 | 0 | 1 |
| 44 | 51 | 80 | 1 | 1 | 0 | 3 | 0 | 1 | 0 | 1 | 0 | 0 | 1 | 0 | 0 | 0 | 0 | 1 | 0 | 0 | 0 | 0 | 1 | 0 | 0 | 1 | 0 | 1 |
| 45 | 52 | 78 | 1 | 2 | 1 | 3 | 1 | 0 | 0 | 1 | 0 | 1 | 1 | 0 | 0 | 0 | 0 | 1 | 0 | 0 | 0 | 0 | 1 | 0 | 1 | 0 | 0 | 0 |
| 46 | 53 | 71 | 0 | 1 | 0 | 1 | 1 | 0 | 0 | 0 | 1 | 0 | 1 | 0 | 0 | 0 | 1 | 0 | 0 | 1 | 0 | 0 | 1 | 0 | 0 | 0 | 0 | 0 |
| 47 | 54 | 71 | 0 | 1 | 1 | 4 | 1 | 1 | 0 | 1 | 0 | 0 | 0 | 0 | 1 | 0 | 0 | 0 | 1 | 0 | 0 | 0 | 1 | 0 | 1 | 1 | 1 | 1 |
| 48 | 55 | 88 | 1 | 2 | 1 | 2 | 0 | 1 | 0 | 1 | 1 | 0 | 1 | 1 | 0 | 0 | 0 | 1 | 0 | 0 | 0 | 1 | 0 | 0 | 1 | 0 | 1 | 1 |
| 49 | 56 | 79 | 1 | 3 | 1 | 3 | 1 | 0 | 1 | 0 | 1 | 0 | 0 | 0 | 0 | 0 | 1 | 1 | 0 | 0 | 0 | 1 | 1 | 0 | 0 | 1 | 1 | 0 |
| 50 | 57 | 72 | 0 | 1 | 0 | 2 | 1 | 0 | 0 | 0 | 1 | 0 | 0 | 0 | 1 | 0 | 0 | 0 | 0 | 0 | 0 | 0 | 1 | 0 | 0 | 0 | 0 | 0 |
| 51 | 58 | 80 | 1 | 3 | 1 | 2 | 1 | 0 | 0 | 1 | 0 | 0 | 0 | 0 | 1 | 0 | 1 | 0 | 0 | 0 | 0 | 0 | 1 | 1 | 1 | 0 | 1 | 1 |
| 52 | 59 | 74 | 0 | 3 | 1 | 3 | 1 | 0 | 0 | 1 | 1 | 1 | 0 | 0 | 1 | 0 | 1 | 1 | 0 | 0 | 0 | 1 | 0 | 0 | 1 | 1 | 1 | 1 |
| 53 | 60 | 74 | 0 | 1 | 1 | 3 | 0 | 0 | 0 | 0 | 0 | 1 | 0 | 1 | 0 | 0 | 0 | 0 | 1 | 0 | 1 | 0 | 1 | 0 | 0 | 1 | 1 | 1 |
| 54 | 61 | 70 | 0 | 1 | 1 | 3 | 0 | 0 | 1 | 0 | 0 | 0 | 0 | 1 | 1 | 0 | 0 | 0 | 0 | 1 | 0 | 0 | 1 | 0 | 1 | 1 | 1 | 1 |
| 55 | 62 | 71 | 0 | 2 | 1 | 3 | 1 | 0 | 0 | 0 | 0 | 0 | 0 | 0 | 1 | 0 | 1 | 0 | 0 | 1 | 0 | 1 | 0 | 0 | 1 | 0 | 1 | 1 |
| 56 | 63 | 74 | 0 | 1 | 1 | 3 | 1 | 0 | 0 | 0 | 1 | 1 | 0 | 0 | 1 | 0 | 0 | 0 | 1 | 0 | 0 | 0 | 1 | 0 | 0 | 0 | 1 | 0 |
| 57 | 64 | 72 | 0 | 2 | 0 | 3 | 1 | 0 | 1 | 0 | 1 | 0 | 1 | 0 | 0 | 0 | 0 | 1 | 0 | 0 | 0 | 0 | 1 | 0 | 0 | 0 | 1 | 0 |
| 58 | 65 | 76 | 1 | 3 | 0 | 2 | 1 | 0 | 0 | 1 | 0 | 0 | 0 | 1 | 0 | 0 | 1 | 1 | 0 | 0 | 0 | 0 | 1 | 0 | 0 | 1 | 1 | 0 |
| 59 | 66 | 82 | 1 | 3 | 1 | 2 | 0 | 1 | 0 | 0 | 1 | 0 | 0 | 0 | 1 | 0 | 0 | 0 | 0 | 0 | 0 | 0 | 1 | 0 | 0 | 0 | 1 | 0 |
