# Supplementary material for: Understanding motivations of older women to continue or discontinue breast cancer screening
Source: PLoS One. 2025 Jun 5;20(6):e0319141. doi: 10.1371/journal.pone.0319141 (PMC12140224; doi:10.1371/journal.pone.0319141)
Supplement: S3 Table — (DOCX) [file pone.0319141.s003.docx]

**SUPPORTING INFORMATION**

**TABLE: DATASET: PEOPLE (ROWS) BY THEMES (COLUMNS)**

This table contains the data for all 59 respondents coded as “1” if a respondent mentioned the thematic code or as “0” if a respondent did not mention it

(to preserve anonymity, actual transcripts are not provided.)

| **ID** | **AGE>75** | **SELF-INITIAT** | **CA STORY** | **DR**  **RECOMEND** | **RECENT SCREEN** | **Continue** | **Discontinue** |
| --- | --- | --- | --- | --- | --- | --- | --- |
| 1 | 1 | 0 | 1 | 1 | 1 | 1 | 0 |
| 2 | 0 | 0 | 1 | 1 | 1 | 1 | 0 |
| 3 | 1 | 0 | 1 | 0 | 0 | 0 | 1 |
| 4 | 0 | 1 | 0 | 1 | 1 | 1 | 0 |
| 5 | 1 | 0 | 1 | 0 | 0 | 0 | 1 |
| 6 | 1 | 0 | 1 | 1 | 1 | 0 | 1 |
| 7 | 1 | 0 | 0 | 0 | 1 | 0 | 1 |
| 8 | 0 | 0 | 1 | 1 | 1 | 1 | 0 |
| 9 | 1 | 0 | 0 | 0 | 0 | 0 | 1 |
| 10 | 1 | 1 | 1 | 0 | 1 | 1 | 0 |
| 11 | 1 | 0 | 0 | 1 | 1 | 0 | 1 |
| 12 | 1 | 0 | 1 | 0 | 0 | 0 | 1 |
| 13 | 0 | 0 | 1 | 1 | 1 | 1 | 0 |
| 14 | 1 | 0 | 0 | 0 | 1 | 0 | 1 |
| 15 | 0 | 0 | 0 | 0 | 1 | 0 | 1 |
| 16 | 0 | 0 | 0 | 1 | 1 | 1 | 0 |
| 17 | 1 | 1 | 1 | 0 | 1 | 1 | 0 |
| 18 | 1 | 0 | 0 | 0 | 0 | 0 | 1 |
| 19 | 1 | 0 | 1 | 1 | 1 | 1 | 0 |
| 20 | 0 | 0 | 1 | 1 | 1 | 1 | 0 |
| 21 | 0 | 1 | 1 | 0 | 1 | 1 | 0 |
| 22 | 1 | 1 | 1 | 0 | 1 | 1 | 0 |
| 23 | 1 | 0 | 1 | 1 | 1 | 1 | 0 |
| 24 | 1 | 0 | 1 | 1 | 1 | 1 | 0 |
| 25 | 0 | 0 | 0 | 1 | 1 | 1 | 0 |
| 26 | 1 | 0 | 0 | 0 | 0 | 0 | 1 |
| 27 | 0 | 0 | 0 | 1 | 1 | 1 | 0 |
| 28 | 0 | 0 | 1 | 0 | 0 | 1 | 0 |
| 29 | 1 | 0 | 1 | 1 | 1 | 1 | 0 |
| 30 | 1 | 0 | 1 | 0 | 1 | 0 | 1 |
| 31 | 1 | 0 | 0 | 0 | 0 | 0 | 1 |
| 32 | 0 | 1 | 1 | 0 | 0 | 0 | 1 |
| 33 | 0 | 0 | 0 | 1 | 1 | 1 | 0 |
| 34 | 0 | 0 | 0 | 1 | 0 | 0 | 1 |
| 35 | 1 | 0 | 0 | 0 | 0 | 0 | 1 |
| 36 | 1 | 0 | 1 | 1 | 1 | 1 | 0 |
| 37 | 0 | 0 | 1 | 0 | 0 | 0 | 1 |
| 38 | 1 | 0 | 0 | 0 | 0 | 0 | 1 |
| 39 | 0 | 0 | 0 | 1 | 1 | 1 | 0 |
| 40 | 0 | 0 | 1 | 0 | 0 | 0 | 1 |
| 41 | 0 | 0 | 1 | 1 | 1 | 1 | 0 |
| 42 | 1 | 1 | 1 | 0 | 1 | 1 | 0 |
| 43 | 0 | 1 | 1 | 0 | 0 | 1 | 0 |
| 44 | 1 | 0 | 0 | 1 | 0 | 1 | 0 |
| 45 | 1 | 0 | 1 | 0 | 0 | 0 | 1 |
| 46 | 0 | 0 | 0 | 0 | 0 | 0 | 1 |
| 47 | 0 | 0 | 1 | 1 | 1 | 1 | 0 |
| 48 | 1 | 1 | 1 | 0 | 1 | 1 | 0 |
| 49 | 1 | 1 | 0 | 1 | 1 | 0 | 1 |
| 50 | 0 | 0 | 0 | 0 | 0 | 0 | 1 |
| 51 | 1 | 0 | 1 | 0 | 1 | 1 | 0 |
| 52 | 0 | 1 | 1 | 1 | 1 | 1 | 0 |
| 53 | 0 | 0 | 0 | 1 | 1 | 1 | 0 |
| 54 | 0 | 0 | 1 | 1 | 1 | 1 | 0 |
| 55 | 0 | 1 | 1 | 0 | 1 | 1 | 0 |
| 56 | 0 | 0 | 0 | 0 | 1 | 0 | 1 |
| 57 | 0 | 0 | 0 | 0 | 1 | 0 | 1 |
| 58 | 1 | 0 | 0 | 1 | 1 | 0 | 1 |
| 59 | 1 | 0 | 0 | 0 | 1 | 0 | 1 |
